# A structure filter for the Eukaryotic Linear Motif Resource

**DOI:** 10.1186/1471-2105-10-351

**Published:** 2009-10-24

**Authors:** Allegra Via, Cathryn M Gould, Christine Gemünd, Toby J Gibson, Manuela Helmer-Citterich

**Affiliations:** 1 Center for Molecular Bioinformatics, Department of Biology, University of Rome Tor Vergata, Via della Ricerca Scientifica, Rome, Italy; 2 European Molecular Biology Laboratory, Postfach 10.2209, 69012 Heidelberg, Germany; 3 Cellzome AG, Heidelberg, Germany; 4 Current address: Biocomputing group, Department of Biochemical Science, Sapienza University of Rome, P.le Aldo Moro 5, Rome, Italy

## Abstract

**Background:**

Many proteins are highly modular, being assembled from globular domains and segments of natively disordered polypeptides. Linear motifs, short sequence modules functioning independently of protein tertiary structure, are most abundant in natively disordered polypeptides but are also found in accessible parts of globular domains, such as exposed loops. The prediction of novel occurrences of known linear motifs attempts the difficult task of distinguishing functional matches from stochastically occurring non-functional matches. Although functionality can only be confirmed experimentally, confidence in a putative motif is increased if a motif exhibits attributes associated with functional instances such as occurrence in the correct taxonomic range, cellular compartment, conservation in homologues and accessibility to interacting partners. Several tools now use these attributes to classify putative motifs based on confidence of functionality.

**Results:**

Current methods assessing motif accessibility do not consider much of the information available, either predicting accessibility from primary sequence or regarding any motif occurring in a globular region as low confidence. We present a method considering accessibility and secondary structural context derived from experimentally solved protein structures to rectify this situation. Putatively functional motif occurrences are mapped onto a representative domain, given that a high quality reference SCOP domain structure is available for the protein itself or a close relative. Candidate motifs can then be scored for solvent-accessibility and secondary structure context. The scores are calibrated on a benchmark set of experimentally verified motif instances compared with a set of random matches. A combined score yields 3-fold enrichment for functional motifs assigned to high confidence classifications and 2.5-fold enrichment for random motifs assigned to low confidence classifications. The structure filter is implemented as a pipeline with both a graphical interface via the ELM resource  and through a Web Service protocol.

**Conclusion:**

New occurrences of known linear motifs require experimental validation as the bioinformatics tools currently have limited reliability. The ELM structure filter will aid users assessing candidate motifs presenting in globular structural regions. Most importantly, it will help users to decide whether to expend their valuable time and resources on experimental testing of interesting motif candidates.

## Background

In recent years it has become clear that proteins with highly modular architectures possess numerous short peptide motifs that are essential to their function [1-5]. Such peptides are termed Linear Motifs (LM) as, in contrast to the globular domains, their function is independent of tertiary structure and encoded solely by the amino acid sequence. They are found in a diverse range of proteins, such as membrane receptors, adaptors, scaffolds and transcription factors, and mediate numerous tasks, which can be as disparate as directing subcellular localization or acting as sites of cleavage. Well-known LMs include peptides binding SH3, Cyclin, PDZ and WW domains [6-10] and phosphorylated peptides interacting with SH2, PTB, BRCT and FHA phosphopeptide-binding domains [11-17]. The biological properties and range of functions mediated by LMs are reviewed in detail elsewhere [4,18-20].

In order to deconvolute the functional components of modular protein architectures, it is necessary to identify the set of LMs as well as the folded components. However, this is not straightforward because simple searches with short sequence patterns, known to act as functional modules, are uninformative - returning a flood of false positive matches. Several tools have been developed to rank motifs based on confidence of functionality by classifying putative motifs based on the hypothesis that functional motifs will have attributes similar to experimentally discovered motifs. Although classification tools cannot definitely confirm a motif as functional (only experimental analysis can achieve this) they can be used to attach a level of confidence to a motif. For example motifs which occur in an incorrect cellular compartment, or outside the known taxonomic range, are unlikely to be functional as are those which are not conserved in closely related proteins or buried in a globular domain inaccessible for interaction. Available motif discovery tools vary in their implementation of confidence-related metrics. ScanProsite [21], the web-based tool for detecting PROSITE [22] signature matches in protein sequences, recently integrated ProRules [23], a database containing additional information about PROSITE profiles, with the aim of increasing the discriminatory power of PROSITE profiles to facilitate function determination and provide biologically relevant information for the annotation of proteins. MnM [24,25], a motif database and a web-based tool for identifying candidate motif occurrences in proteins, addresses the issue of non-functional false positives by implementing evolutionary conservation, surface prediction and frequency scores to rank motif occurrences in a protein query. The Eukaryotic Linear Motif (ELM) resource filters implausible motif occurrences according to cell compartment and taxonomic range [2]. It also indicates less likely matches that lie within globular domains annotated in the SMART [26] and Pfam [27] resources and contrasts these with intrinsically unstructured polypeptide (IUP) regions predicted by GlobPlot [28] that are more likely to be motif-rich [5]. DILIMOT and SLiMFinder - tools designed for discovery of candidate novel peptide patterns significantly enriched in protein interaction datasets - also use some of these techniques to improve confidence in returned motifs [29,30]. Sequence conservation has also been shown to be effective in up-weighting true motifs relative to false positive matches [31-33].

In the intracellular milieu, LMs are found to be particularly abundant in segments of IUP where they are readily accessible [34]. Accessibility is a basic requirement of LM function which is almost always mediated by direct interaction with globular domain ligands. Extracellular proteins tend to have much less natively disordered polypeptide and therefore the extracellular linear motifs such as N-glycosylation sites [35] and the integrin-binding RGD motif [36] usually occur within globular domains, most often residing in exposed loop regions. LMs are also regularly found in globular regions of intracellular proteins - for example phosphorylation sites are common in flexible loops [37]. However, close inspection of the literature also reveals many instances of candidate motifs falsely reported as functional on the basis of loss of function mutagenesis and out-of-context peptide-binding experiments, despite the motif being well structured and sometimes deeply buried in a globular domain [38-41].

This observation suggests that stringent examination of motif structural context should be an essential processing step for experimental analysis. It also advocates the importance of high quality tools to identify such cases, as the cost associated with failure is detrimental both in terms of effort and quality of the literature. Despite this, neither the ELM globular domain classification nor the MnM surface prediction score take advantage of all the information available to them in the form of the plethora of experimentally solved protein structures. ELM globular domain classification is overly strict, classifying motifs occurring in these regions as low confidence. The MnM surface prediction score uses primary sequence based prediction both in those cases where a structure is available and in regions where a disorder predictor will render secondary structure prediction unnecessary.

In the present manuscript, we address the issue of LM accessibility when the matches occur within globular domains for which a reference three-dimensional (3D) structure is available. Development and calibration of a structure filter is currently not straightforward as there are relatively few available structures for most motif classes (an obvious exception being N-glycosylation sites), placing limitations on the training and benchmarking possibilities. Nevertheless, we have been able to develop a protocol in which reference domain structures are selected and then the matched motifs evaluated using accessibility and secondary structure parameters.

Benchmarking of the structure filter suggests that deeply buried LM candidates are unlikely to be functional, and that the likelihood of motif matches being valid functional sites improves with accessibility. In this way, the new filter can aid researchers to decide whether they wish to invest effort inexperimentaltesting of candidate motifs. The structure filter pipeline is implemented in a publicly available Python program accessible via a web-service interface [42]. The structure filter is fully integrated into the ELM server [43], providing graphical representation of the results in the context of the other filters.

## Results

### The ELM structure filter scoring scheme

**Figure 1:**
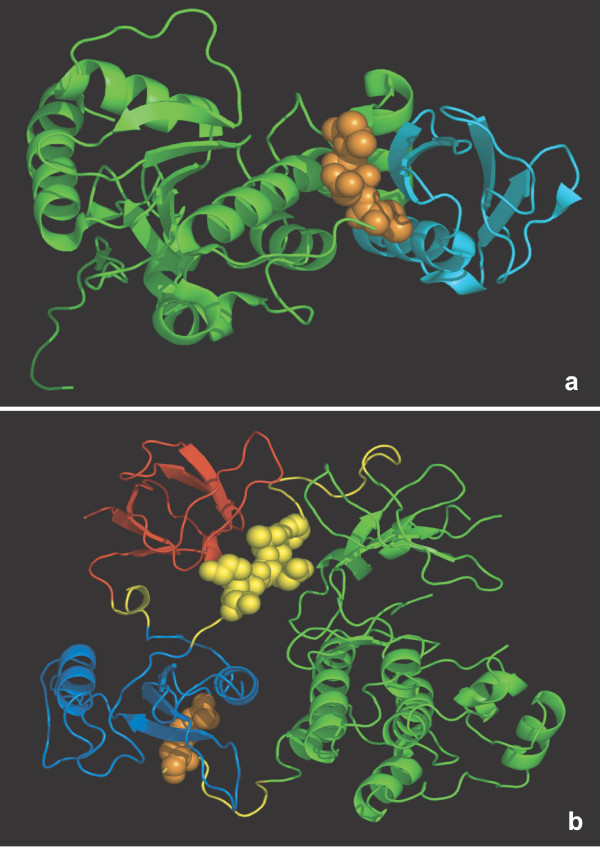
**Two examples of linear motifs packed into structured domains**. a) PDB 2D07: Sumo-interacting motif (orange) of TDG domain (green) bound to SUMO-3 protein (cyan); b) PDB 2PTK: closed conformation of the proto-oncogene tyrosine-protein kinase Src. Blue: SH2 domain; red: SH3 domain; green: protein kinase; orange: pTyr-527; yellow: linkers; yellow spheres: SH3 binding peptide. All structure views were prepared with PyMOL .

**Figure 2:**
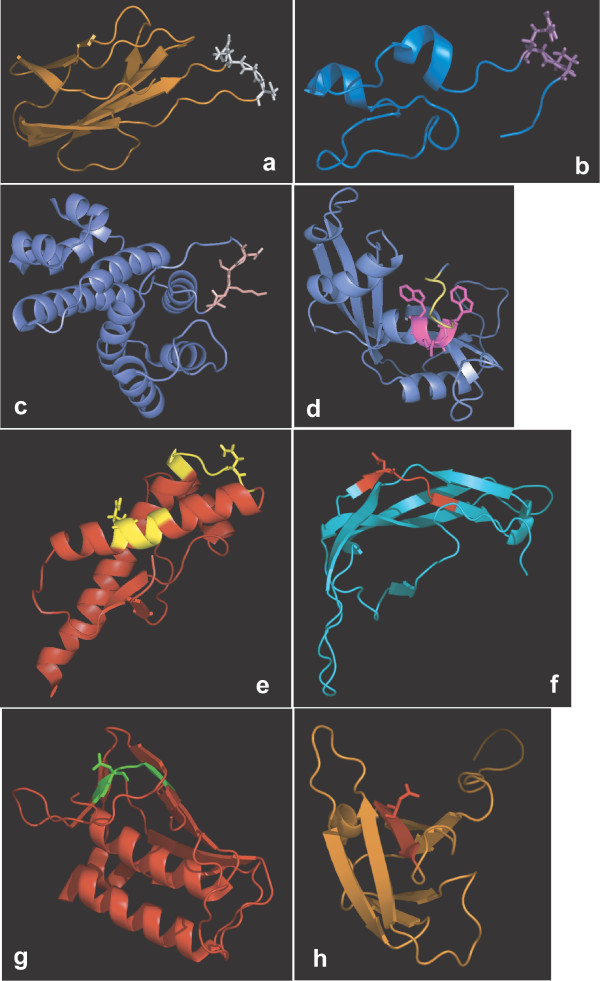
**Examples of linear motifs with functional residues protruding outwards from the structural domain surface**. a) A very exposed instance (in white) of LIG_RGD in a loop of SCOP domain d1 mfn_2; b) An instance (in violet) of LIG_RGD in a region outside a domain (SCOP d1ssua_); c) An instance (in pink) of MOD_SUMO in an exposed loop of the d1kpsd_ SCOP domain; d) The MOD_CMANNOS C-Mannosylation site (in magenta) in the SCOP domain d1k2aa_; e) The two MOD_N-GLC_1 N-glycosylation sites (in yellow) in the SCOP domain d1qm3a_; f) The N-glycosylation site (in red) in the SCOP domain d1fl7b_; g), h) The N-glycosylation site (in green) in the SCOP domain d1o7ae2 and (in red) in the SCOP domain d1n26a1.

**Figure 3:**
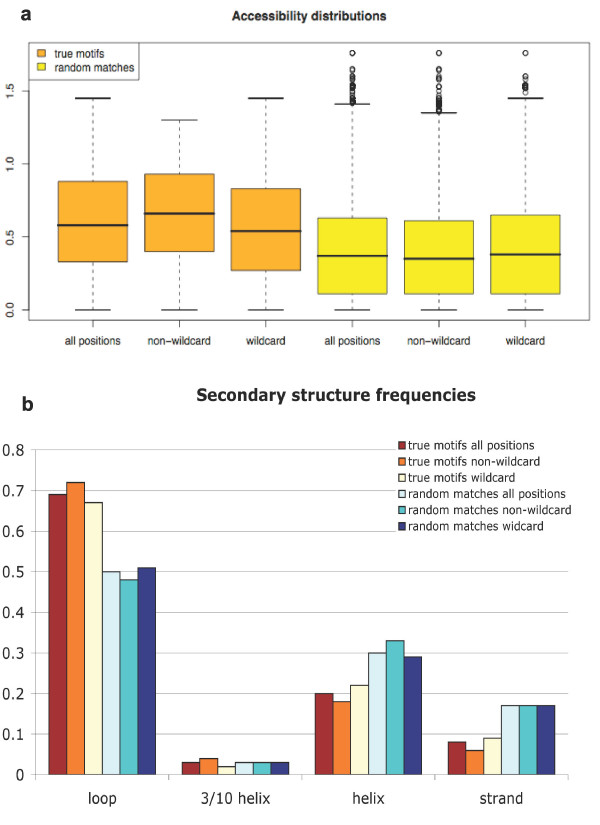
**Secondary structure frequency and accessibility distribution for true motif instances and for random matches**. **3a**) Boxplots representing the accessibility distributions of true motif instances (orange) and of random matches (yellow), calculated for all motif positions (all positions), non-wildcard positions only (non-wildcard) and wildcard positions only (wildcard). The solid box lower and upper bounds represent the 25th and 75th percentile, respectively. Circles represent outliers; **3b**) frequencies of each secondary structure element type in true motif instances and in random matches, calculated for all motif positions, for non-wildcard positions only and for wildcard positions only.

Structural analysis of true motif instances annotated in ELM supported what is expected from LM biology [3], *i.e*. that they tend to lie on the surface of protein domains and prefer unstructured and loop regions (See below "Analysis of the ELM 3D benchmarking dataset"). Figure 1 shows two examples of motifs lying on domain interfaces whereas Figure 2 reports cases of motif instances whose functional residues protrude outwards from the domain surface and hence are accessible to the solvent. This observation was further supported by the comparison between the accessibility and secondary structure distributions of true motifs *vs *random matches (determined as described in Methods) in our datasets (Figure 3), which highlights that true motifs are on average more accessible than random matches (p-value = 1.9e-55); moreover, loops are more represented (p-value = 1.13e-35) in true motifs than in random matches and both alpha-helices and strands are less represented in true motifs than in random matches (p-value = 3.69e-12 and 2.66e-16, respectively). These results convinced us to base the structure filter scoring scheme on accessibility and secondary structure assignments.

The aim of the scoring procedure is to assign a score to LM candidates in the user query sequence given that a reference structure is available. In order to do this, the structure filter scans the LM match 3D context position by position, evaluates the relative accessibility and the secondary structure of each single position *i*, and assigns an accessibility score (*Q*_*acc*_) and a secondary structure score (*Q*_*sse*_) to the motif match as the normalized sums of its single position scores.

More specifically, the score of a motif match is calculated on the non-wildcard positions of the regular expression pattern for the motif as:



where *N *is the number of non-wildcard positions of a match, i.e. the number of non-wildcard residues in a LM occurrence, *i *is the *i*^th ^position along the match, *i *∈ Ω means that the sum is limited to the set of non-wildcard positions, Ω, and *q*(*i*) is the positional score of position *i*. Note that *Q*_*acc *_and *Q*_*sse *_were also calculated for all LM positions (i.e. not limiting the sum to the set of non-wildcard positions) and found to be marginally less discriminating than those only based on non-wildcard positions. In this regard, Figure 3 shows that the accessibility differences between wildcard and non-wildcard positions are statistically significant in the case of true motifs (t-test's confidence level = 0.99, p-value = 3.058e-05) (Figure 3a) and that true motif non-wildcard positions have a more pronounced tendency to be in loops and a less marked disposition to be in helices and strands as opposed to the frequencies both of true motifs for all positions and for wildcard positions, even if none of these differences is statistically significant (Figure 3b). For further details see additional file 1, additional file 2 (Figure S1) and additional file 3 (Figure S2).

We adopted as accessibility positional score, *q*_*acc*_(*i*), of position *i*, the normalized solvent exposure value of the residue in *i*, which ranges between 0 (non exposed) and 1.5. Thus, the higher the residue exposure, the more the corresponding position is rewarded. The secondary structure positional score, *q*_*sse*_(*i*), was determined in a more complex manner. The analysis of LM instances on structural domains showed that they occur more frequently in loops and unstructured regions than expected by chance. In order to quantify this observation, we calculated, for each secondary structure element (SSE) type (loop, helix, strand, 3/10 helix - see Methods), the ratio between the SSE type frequency (**ν**) among true motif instances and among random matches. The corresponding values are reported in Table 1.

**Table 1:** Frequency of secondary structure elements in true and in random motifs

		**loop**	**3/10 helix**	**helix**	**strand**
**νTM**		0.72	0.04	0.18	0.06

**ν Random**		0.48	0.03	0.33	0.17

**νTM/νRandom**	*q*_*sse*_*(i)*	1.50	1.33	0.55	0.35

The table reports the secondary structure type frequencies observed in motifs of the benchmark and random datasets calculated considering only non-wildcard positions. **νTM**: frequency in the true motif instance dataset; **νRandom**: frequency in the random match dataset; *q*_*sse*_*(i) *= secondary structure score of position *i*.

Thus, the secondary structure score of a position *i *whose SSE assignment is *loop *(or *3/10 helix*, *helix*, *strand*), is the ratio between the frequency of loops (or 3/10 helices, helices, strands) in the instance dataset and the frequency of loops (or 3/10 helices, helices, strands) in the random dataset.

### Assessing the predictive ability of the ELM structure filter

In order to assess the predictive ability of the ELM structure filter scoring scheme, we made use of five strategies, each introducing useful parameters for the evaluation of the discrimination power of our procedure: 1) we plotted ROC curves and calculated AUCs; 2) we assigned a p-value to predictions; 3) we built LM-specific background distributions; 4) we identified sparse/neutral/enriched score intervals; 5) we carried out a 5-fold cross validation in order to determine sensitivity, specificity and accuracy.

In order that the structure filter may be a useful guide to the ELM resource user, we propose that the values of the above-mentioned parameters are used as decision-making tools in evaluating the score of LM predictions. In particular, since having high accessibility and belonging to loop regions is not a prerogative of LMs alone and the random match dataset might in principle be "contaminated" by not yet annotated spurious true motifs, we suggest using as many indicators as possible in evaluating a prediction score and not relying on each single tool as a unique criterion for retaining/rejecting a prediction.

#### 1) ROC curves and AUCs

**Figure 4:**
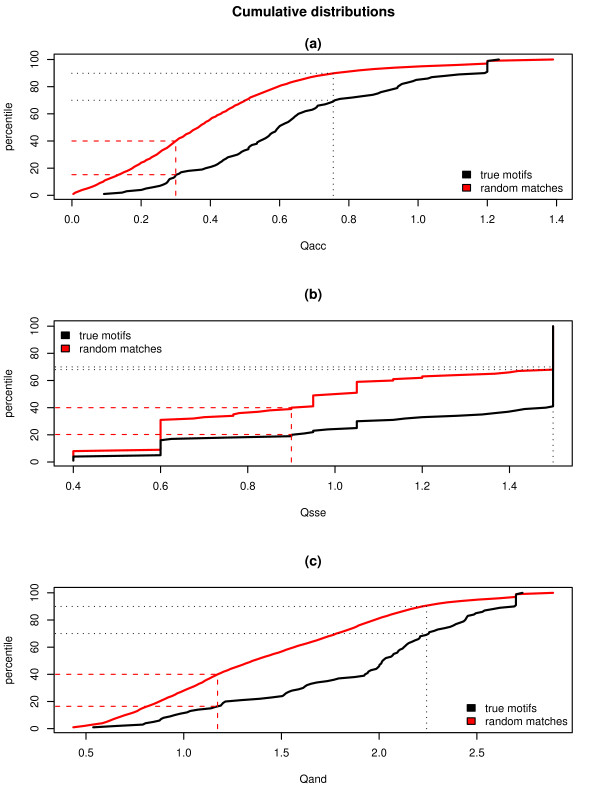
**Cumulative score distributions**. a) The cumulative distribution of (a) Q_acc_, (b) Q_sse_, and (c) Q_and _= Q_acc _+ Q_sse _scores calculated for true motif matches (true motifs), and for random matches (random matches) in non-wildcard positions. Red dashed lines indicate the percentile cut-off ensuring that the lower 40% random matches fall in the "sparse" bin. The consequent percentage of true motifs falling in the "sparse" bin is about 15% (accessibility) and 20% (secondary structure). This cut-off corresponds to *Q*_*acc*_~0.3 and *Q*_*sse*_~0.7. Black dotted lines indicate the percentile cut-off that guarantees that the enriched bin collects at least the top 30% true motifs. This cut-off corresponds to *Q*_*acc *_= 0.76, *Q*_*sse *_= 1.5, and *Q*_*and *_= 2.243.

**Figure 5:**
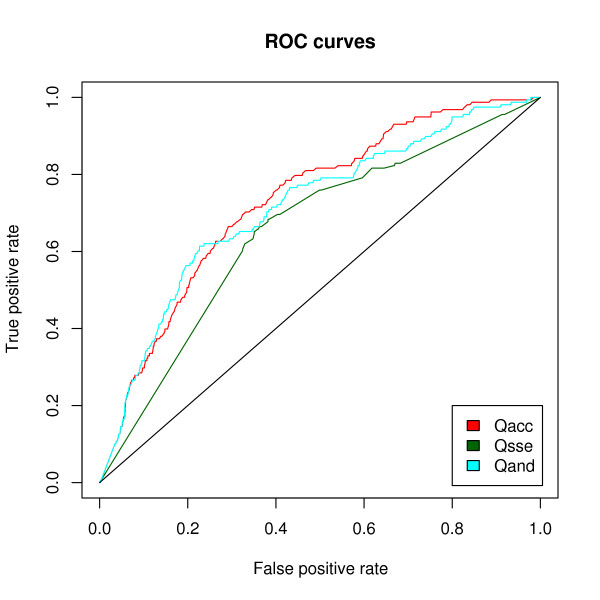
***Q*_*acc*_, *Q*_*sse *_and *Q*_*and *_= *Q*_*acc*_+ *Q*_*sse *_ROC curves**.

In order to establish if one score is more discriminative than the others, we assigned an accessibility score (*Q*_*acc*_), a secondary structure score (*Q*_*sse*_) and a combined score (*Q*_*and *_= *Q*_*acc *_+ *Q*_*sse*_) to the true positive instances of our dataset and to the random matches of the random dataset, plotted cumulative score distributions and ROC curves and calculated the area under the ROC curves (AUCs). In calculating the ROC curves, we assumed that random matches are all negative matches. Figure 4 shows that the cumulative distribution of true motifs is clearly separated from that of random matches for each score type. Moreover, the ROC curves (Figure 5) show that all three score types are able to discriminate between the true motif and random match sets and that both *Q*_*acc *_and *Q*_*and *_perform better than *Q*_*sse*_; the AUC values for the three scores are 0.73 (*Q*_*acc*_), 0.66 (*Q*_*sse*_) and 0.72 (*Q*_*and*_); notice that, even though the AUC for *Q*_*and *_is slightly lower than that of *Q*_*acc*_, *Q*_*and *_performs similarly or better than *Q*_*acc *_in the range corresponding to the 20% of the ROC x-axis values.

#### 2) p-value

We determined the distribution of random matches and use it to assign a p-value to the score of each ELM prediction. This p-value, which is implemented both in the Web Server and in the Web Service, is calculated using a Z-test and is a conservative estimate of the probability that a LM prediction with a given score is a true positive; more specifically it is the probability of obtaining a random match with a score at least as high as the one that was actually observed, and therefore we expect it to be very stringent.

#### 3) LM-specific background score distributions

Due to the paucity of true motif instance data, we cannot build a true motif score distribution for each ELM motif (and therefore we cannot build a LM-specific structure filter yet) and compare it to the corresponding random motif score distribution. However, we built, and displayed in the ELM web server output page, LM-specific random score distributions (as described in Methods) in order to use them as background score contexts, telling the users something about the average behavior (in terms of accessibility, secondary structure and combined scores), on a large dataset of structures, of each single LM. These background distributions are only intended as a supplementary guideline for the web users to evaluate whether or not the score assigned to a LM match is reasonably higher than the random match score average for that LM.

The background score distributions for 103/112 motifs are shown in the additional file 4 (Figure S3).Correspondences between x-axis labels and ELM names are reported in the additional file 5

#### 4) Sparse/neutral/enriched score intervals

We chose two score thresholds for each score type aimed at defining three score intervals (or "bins"), one "sparse", lacking in true motifs and enriched in random matches, one identifying "neutral" matches, and one lacking in random matches and enriched in true motifs. We consider that such a three-interval scheme might effectively help the user in deciding whether to retain or reject a prediction. In fact it is based on the idea that a predicted match that is assigned a score in the "enriched" interval will be indicated by our procedure as a good true motif candidate (i.e. likely to be a valid functional site), motif matches scoring in the bottom interval ("sparse" interval) as unlikely to be valid functional sites and those ranking in the middle one as "neutral". The score thresholds were chosen on the basis of the cumulative distributions of Figure 4 by selecting two cut-offs (one in the percentile range 0-50% and one in the percentile range 50%-100%), roughly corresponding to the inflection points of the random match cumulative distributions, and guaranteeing that at least the top 30% true motifs are retained in the enriched bin and at least the lower 40% random matches fall in the sparse bin. The "neutral" bin is delimited by the "sparse" and "enriched" cut-offs and contains the medium quality matches. Table 2 reports *Q*_*acc*_, *Q*_*sse *_and *Q*_*and *_thresholds defining the three bins. From Figure 4 and Table 3, it can be seen that, in the case of the accessibility score (Figure 4a), the cut-off on the top 30% of true motifs implies that only 10% of random matches are retained in the enriched bin and that the cut-off on the lower 40% random matches implies that only 15% true motifs incorrectly fall in the "sparse" bin. In contrast, *Q*_*sse *_thresholds (Figure 4b) actually assign about the top 60% true motifs and 32% random matches to the enriched bin (see Table 3). This is due to the fact that the top 60% true motifs (and 32% random matches) uniformly get the highest score. Finally, in the case of *Q*_*and *_(Figure 4c), only 9% random matches are retained in the enriched bin and only 16% of true motifs fall in the sparse bin (Table 3). This gives to the users a measure of the percentage of false hits that they can expect in the enriched bin and of the percentage of true hits that they would miss if discarding all the predictions falling in the sparse interval.

**Table 2:** Score thresholds defining the "sparse", "neutral" and "enriched" bins

**Bin**	***Q*_*acc*_**	***Q*_*sse*_**	***Q*_*and*_**
sparse	≤0.3	≤0.9	≤1.173

neutral	> 0.30 and < 0.755	> 0.9 and < 1.5	> 1.173 and < 2.243

enriched	≥0.755	≥1.5	≥2.243

*Q*_*acc*_: accessibility score; *Q*_*sse*_: secondary structure score; *Q*_*and *_= *Q*_*acc *_+ *Q*_*sse *_.

**Table 3:** Number and percentage of true and random motifs assigned to each bin by the different score types

**Score type**	**bin**	**TM**	**%TM**	**random**	**%random**	**ratio**
*Q*_*acc*_	sparse	24	15.19	8835	40.05	0.3793
	
	neutral	86	54.43	11106	50.34	1.0812
	
	enriched	48	30.38	2119	9.61	3.1613

*Q*_*sse*_	sparse	33	20.89	8910	40.39	0.5172
	
	neutral	30	18.99	6004	27.22	0.6976
	
	enriched	95	60.13	7146	32.39	1.8564

***Q*_*and*_**	**sparse**	**26**	**16.46**	**8821**	**39.99**	**0.4116**
	
	**neutral**	**84**	**53.16**	**11263**	**51.06**	**1.0411**
	
	**enriched**	**48**	**30.38**	**1976**	**8.96**	**3.3906**

**Score type**: can be based on accessibility (*Q*_*acc*_) only, on secondary structure only (*Q*_*sse*_) or on a combination of them (*Q*_*and *_= *Q*_*acc *_+ *Q*_*sse*_); **TM (random)**: number of sparse/neutral/enriched true motif (random) matches; **%TM (%random)**: percentage of sparse/neutral/enriched true motif (random) matches; **ratio**: %TM/%random. The scoring scheme implemented both in the Web Server and in the Web Service is marked in bold.

#### 5) 5-fold cross validation

In order to establish more rigorously the predictive ability of the structure filter in the enriched and sparse intervals, we carried out a 5-fold cross validation experiment. Referring to score calibration and within the limits of the 5-fold cross validation experiment only, we defined two intervals instead of the three implemented in the ELM Web Server, by incorporating the neutral interval first into the enriched one and then into the sparse one. This made it possible to properly determine sensitivity and specificity values in two different situations: the first accounting for an enrichment of sensitivity and the second for an enrichment of specificity.

We defined the positive dataset as the one made up of the ELM true instances and the negative dataset as the set of all the un-annotated random matches. We split both the positive and the negative datasets into five subsets by random sampling the datasets without replacement, thus obtaining five non-overlapping positive and five non-overlapping negative training sets. Five positive (negative) test sets were determined by depriving cyclically the whole positive (negative) dataset of each of the five positive (negative) training sets.

We built the scoring schemes as described in the section "The ELM structure filter scoring scheme" and set up score acceptance/rejection thresholds on the training sets as explained above (subsection "Sparse/neutral/enriched score intervals"). Then, we validated them on the corresponding test sets by calculating sensitivity (S_n_), specificity (S_p_), and accuracy defined as:



In evaluating *S*_*n*_, *S*_*p *_and *Accuracy*, we assumed that a match belonging to the negative set and scoring above the "accept" threshold, is a FP and one scoring below, is a TN; a true instance scoring above the "accept" threshold is a TP and one scoring below is a FN.

Sensitivity (*S*_*n*_) and specificity (*S*_*p*_) and accuracy averaged over the five sets are reported in Table 4. Since the structure filter is designed as a guide to experimentation, we consider that sensitivity should be privileged over specificity - for not missing too many true motifs. Based on this viewpoint, it can be observed in Tables 3 (last column) and 4 that the best performing scoring schemes - in terms of a trade-off between sensitivity, specificity, the percentage of true motifs erroneously discarded and the percentage of true motifs correctly retained - are *Q*_*acc *_and *Q*_*and*_.

**Table 4:** Sensitivity, specificity and accuracy obtained with the 5-fold cross validation experiment.

**score type**	**<S_n_>**	**<S_p_>**	**<Accuracy>**
	*enriched + neutral merged*		

*Qacc*	0.843 ± 0.017	0.400 ± 0.003	0.404 ± 0.003

*Qsse*	0.780 ± 0.019	0.422 ± 0.022	0.425 ± 0.022

*Qand*	0.818 ± 0.018	0.399 ± 0.003	0.402 ± 0.003

	*sparse + neutral merged*		

*Qacc*	0.295 ± 0.052	0.907 ± 0.006	0.902 ± 0.005

*Qsse*	0.61 ± 0.025	0.662 ± 0.01	0.661 ± 0.01

*Qand*	0.288 ± 0.049	0.911 ± 0.004	0.907 ± 0.004

Sensitivity, specificity and accuracy are averaged over the five datasets defined in the 5-fold cross validation experiment for: a) the neutral interval incorporated into the enriched one and b) the neutral interval incorporated into the sparse one. <S_n_>: average sensitivity; <S_p_>: average specificity; <Accuracy>: average accuracy.

Notice that the *Accuracy *values reported in Table 4 might be affected by the fact that the positive and negative datasets are unbalanced.

The analysis of the ROC curves, of the cumulative distributions and of the filter performance in the three score bins suggests a more relevant role of the accessibility in discriminating true from false motifs than the secondary structure assignment. This observation is biologically sound since, while a buried motif is unlikely to be a genuine functional site, an exposed motif lying e.g. on a helix can in any case possess an interaction ability. Finally, our results show that the combined score is slightly more effective than the accessibility score and markedly better than the secondary structure score. The combined score *Q*_*and *_is implemented in both the Web Server and Web Service.

### Usage of the ELM structure filter

For practical purposes, the filter exploits available information on protein structures to answer the question "Is it worth testing this motif candidate experimentally?" rather than to categorically tell the users whether they have a real motif or not.

In deciding if a prediction is a good experimental candidate, the user should give more weight to accessibility score than to secondary structure score since a buried motif is unlikely to carry a function, whereas an exposed motif may function properly even if it is part of a beta strand or belongs to a helix (see examples in the benchmarking dataset, additional file 6 (Table S1).

The main exception to well buried candidates being nonfunctional concerns allosteric rearrangements [44]. If the motif is in the core of a well-known domain like SH3 or a TIM barrel, a review of the accumulated structural knowledge will allow the user to conclude that the chance of valid function is negligible. If there is evidence of allostery, however, depending on which parts of the structure are flexible, this might support or invalidate the motif. If nothing is known, then it should be kept in mind that most parts of most globular domains do not undergo major rearrangements, hence candidates from the sparse bin should not be eyed with hope.

The user should also consider overall context in assessing the structure filter results. Is the cell compartment correct: An exposed RGD motif with a significant p-value in an extracellular protein is a very good integrin-binding candidate: one in a nuclear protein is worthless. Is the motif conserved, at least within a phylogenetic lineage such as mammals, tetrapods or vertebrates: the motif should be conserved in such groups if it is functional in a regulatory system common to related organisms. Is the biological context sensible: Is the query protein in some way functionally associated with the ligand protein; Are they in the same regulatory pathway; Are they in the same protein complex?

### Structural analysis of LMs: Classification and examples of motifs in protein structures

#### Globular Domains as the structural unit for LM evaluation

Before the structural context of LMs can be evaluated, it is necessary to define and select the structural unit. Structure files may contain large protein complexes, single proteins, single or multiple chains, single globular domains and many other types of molecule. LMs may be bound to their ligands or in an unliganded state. Figure 1a shows the Sumo-Interacting Motif (SIM) of TDG bound to SUMO-3 by beta augmentation but also well packed into the main TDG domain. Clearly we need to measure accessibility of the SIM in the absence of the SUMO protein. The open (active) and closed (inactive) conformations of the Src kinase are dependent on the phosphorylation states of several tyrosines. In particular, the closed conformation is specified by an interaction between the Src SH2 domain and the C-terminal pTyr-527 and an interaction between the Src SH3 domain with a peptide linking the SH2 and kinase domains. Figure 1b shows the closed conformation with these elements highlighted. In particular, the SH3 binding peptide is fully buried, even though it is not part of a globular domain. In the open conformation this peptide is much more accessible, as is the C-terminal peptide which is released from the SH2 domain (e.g. 1Y57, [45]). The dependency of LM accessibility on globular domain rearrangements implies that multi-domain structures are not a suitable structural unit for structure filtering. The appropriate units therefore in the cases of LMs would be the individual globular domains themselves. At least for domains that do not undergo allosteric rearrangement, a motif which is buried in the core of a structural domain unit is unlikely to be a true one. Therefore we chose the SCOP [46] protein domain definition as provided by the ASTRAL resource [47] as the structure dataset to be used to implement the structure filter.

#### Analysis of the ELM 3D benchmarking dataset

The inception of this work required the collection and analysis of the 3D occurrences of LM instances annotated in the ELM resource [1]. Here we present a discussion of our benchmark dataset. Many details and specific examples are reported in the supplementary information (additional file 1). As described in Methods, we obtained a set of 158 3D non-redundant instances from 36 different LM entries (reported in additional file 6 (Table S1) from the ELM resource release June 2007. Sixteen motifs match only one instance and twenty match two or more.

##### Motifs in loops and flexible regions

The majority (~60%) of LM instances are made up of residues whose relative accessibility to the solvent is at least 50% and are located entirely in loop, turn or unstructured regions. Figure 2 shows two typical examples (LIG_RGD and MOD_SUMO) of a motif in a very exposed loop of a domain (2a and 2c) and a motif in a flexible region which is not in a domain (LIG_RGD, Figure 2b). LIG_RGD is a short peptide ligand motif which interacts directly with extracellular domains of integrins whereas MOD_SUMO is a motif recognised for modification by SUMO-1. The SUMO proteins are **S**mall **U**biquitin-related **MO**difiers that are covalently conjugated onto lysine residues within target sequences.

##### Motifs in more structured regions

Eight out of 36 LM entries have at least one instance which is entirely or almost entirely in helical conformation while two entries have at least one instance almost entirely in a strand conformation. Notwithstanding the greater rigidity of helices as opposed to loops and unstructured regions, LMs found in helical conformation are not necessarily prevented from being exposed to the solvent and carrying out their functions. Two clear examples are shown in Figure 2d and 2e. Figure 2d shows an instance of the MOD_CMANNOS motif, which is part of a helix and is partly hidden by the C-term of the protein. C-Mannosylation is a type of protein glycosylation involving the attachment of a mannosyl residue to a tryptophan. In this particular case, the most buried residues are those corresponding to wildcard positions in the MOD_MANNOS regular expression (W..W), whereas the conserved tryptophan needed for the mannose attachment is protruding outwards from the domain surface. Figure 2e reports two MOD_N-GLC_1 N-glycosylation sites on the same domain. N-linked glycosylation is a co-translational process involving the transfer of an oligosaccharide chain to an asparagine residue in the protein. In this case, one site is part of a well exposed helix whereas the other one consists of a loop with small helix overlap and it is very exposed.

Figure 2f, g and 2h show cases of LMs in partly buried beta strands. In figure 2f an instance of the MOD_N-GLC_1 motif is in a long edge beta strand, slightly disrupted, and quite exposed. The N-glycosylation sites of figures 2g and 2h are two examples of motifs lying on partially hidden beta strands but whose modified asparagine (involved in the functional activity) side chain is exposed to the solvent.

##### Motifs with low accessibility

In our benchmarking dataset, 29/158 instances belonging to 11 different LMs [marked in dark orange in additional file 6 (Table S1)] have a very low average accessibility. In 15/29 of these instances, however, residues belonging to non-wildcard positions in the LM regular expression (e.g. the two tryptophans in the C-Mannosylation site regular expression W..W) display equal or higher accessibility values as opposed to wildcard positions (marked in bold in the acc_nwc column of Table S1, additional file 6). This seems reasonable since LMs are involved in protein interactions and the non-wildcard positions specify LM function. Importantly, this trend is not seen in the case of LM false positive matches, an observation which helped us to improve the benchmark set as it brought to light some poorly annotated instances. See additional file 1 for details.

##### Buried motifs

In the benchmark dataset there are a few cases (10/158) of almost completely buried true motif instances, i.e. displaying an average relative accessibility < 0.2 on the non-wildcard positions. We analysed them one by one by manual inspection and concluded that they fall in one of two situations: either their functional residue(s), or at least their side chains, are favourably oriented outwards from the domain surface (see additional file 1, additional file 6, and Figure 2g and 2h), or an allosteric effect is either known or reasonable to hypothesize. Additional file 1 reports details and specific examples.

## Discussion

We have set up a procedure to help in the discrimination of true from false positive LM matches, that is based on the information coming from two important features inherent to the 3D structure of proteins: accessibility to the solvent and secondary structure element. The fact that functional LMs tend to be in flexible and accessible regions of proteins is biologically sound and is furthermore supported by the structural analysis of experimentally validated instances of LMs carried out in this work. As a consequence, our approach will advise a user against considering a match as a true motif if it resides in an unfavourable structural context. Nevertheless, the function of proteins can be regulated by an assortment of different mechanisms, and allosteric modifications or unusual LM position and/or conformation are infrequent but possible. In this sense, we encourage the user to carefully evaluate the possibility that a hidden motif can become exposed upon protein interaction and to use the ELM structure filter *cum grano salis*, i.e. not as a deterministic predictor but rather by exploiting the supplied 3D information on LM predictions as a supplement to a prior knowledge of the LM biological context.

The ELM resource now provides three ways to aid the user about structural context for the query sequence. The disorder predictor GlobPlot highlights potential motif-rich regions that are likely to be intrinsically unstructured. SMART and Pfam domains define regions of well-defined globular structure where LMs are expected to be rare. Where it can be applied, the new structure filter now provides a benchmarked estimate of LM likelihood. MnM has taken a different approach to structural context, a single score for each pattern match being provided by an accessibility prediction algorithm, SPS [24,48]. While MnM does not supply domain and tertiary structural information that is highly informative to the user, an accessibility predictor does have a unique value for a substantial fraction of protein sequence space that is predicted to be globular but is not known to be related to a solved domain structure. In future, we may also consider introducing a predictive accessibility filter into ELM for poorly characterised globular peptide segments. There are many algorithms in the literature, with the current best performing reported to be NetSurfP and Real_SPINE [49,50].

Besides the results on the structure filter discrimination power presented in this work, we want to point out that the process of developing the structure filter has already proven of value to the ELM resource. The structural analysis of annotated motifs reported in section 2.1 highlighted a number of questionable motifs that turned out to be incorrectly annotated with weak or conflicting support in the literature. In this regard, experimentalists should be aware that accurate annotation of LMs concurs with developing effective methodologies aimed at identifying new putative motifs and that inference of shortlists of candidate true motifs is especially useful to reduce the number of assays needed to experimentally validate a new LM. Thus, the experimental strategy adopted to detect functional motifs plays a fundamental role and incorporating some simple stratagems in experimental protocols might crucially help in reducing the number of false motifs in the literature. We consider a pair of much too rarely undertaken controls to be especially important when candidate motifs are mutated [4]: (1) Check if the motif mutation unfolds the protein by cloning in a tagged expression construct that allows fast and easy purification of the protein and examine folding status by e.g. circular dichroism (or NMR if available); (2) When transfecting with mutated proteins, examine the cells by microscopy for intracellular amyloid caused by massive overexpression of unfolded protein and, if it is present, then reason out why the assay is misleading (e.g. remember that amyloids are not subject to ubiquitin-mediated destruction processes so destruction box and degron motif mutation assays give misleading results).

We expect that the predictive power of the structure filter can be improved as more data becomes available. For example, one might devise a procedure trained on the structural data of specific motifs and qualified to make predictions only for those motifs. We investigated this approach and concluded that it would currently be applicable only to the very few LMs that have enough instances in the database. For the great majority of LMs, appropriate training and tests cannot be carried out and predictions turned out to be unacceptably stringent: An effective procedure should be based on many more instances *per *LM and these are not available at the moment. We believe that in the future, as an increasing number of protein structures become available and the quantity of ELM annotation data grows, it will be possible to appropriately train and test motif-specific structure filters for a significant number of LMs.

## Conclusion

In conclusion, LMs are subject to enormous over-prediction, so that the few true motifs are lost amongst the many false positives. Whenever a query can be modelled on a structure, the structure filter can help in discriminating true from false positive matches of LMs. Moreover, since the number of solved structures is rapidly increasing, a benchmark set of true positive structures is going to be available for an increasing number of motifs, thus allowing more reliable tests and consistent score threshold setups. As a consequence, the structure filter, which can be considered to all intents and purposes as a precursor in the use of structural information for short LM false positive discrimination, is going to become increasingly indispensable for the ELM resource's filtering framework in the structural genomics era.

## Methods

### Dataset of structural instances

The ELM database ([1], release June 2007) collected 112 LM, 93 of which have annotated instances. The set of 1898 annotated instances in 1037 sequences from the 93 different LMs obtained from the ELM database represented our initial dataset. In our vocabulary, "instance" means a true annotated LM occurrence, whereas "match" indicates any regular expression hit on a query sequence. The instances were modeled onto SCOP domains [46] by BLAST alignment [51] of the sequence containing the instance to the reference domain sequence extracted from the PDB entry [52]. In order to assign a "sequence instance" to a "structure instance", the aligned sequences must have at least 70% global identity (over the domain) and 100% local identity (i.e. along the instance positions). The final dataset of structural instances comprised of 185 3D instances from 37 different LMs. Redundancy was removed at the structure level: if two or more instances mapped on identical 3D sites, all but one were discarded, thus reducing the dataset to 158 3D instances from 36 different LMs.

For each position of a 3D instance, the solvent accessibility and secondary structure values are collected from the DSSP [53] file of the target structure mapping the instance. For the solvent exposure of a residue, a relative (normalized) value is calculated as the ratio of the residue's DSSP accessibility value to the residue accessible surface area value as defined by Miller and co-workers [54] and which is calculated for the residue in a Gly-Xaa-Gly tripeptide in extended conformation.

The DSSP secondary structure types are: H = alpha helix, B = residue in isolated beta-bridge, E = extended strand (participates in beta ladder), G = 3-helix (3/10 helix), I = 5 helix (pi helix), T = hydrogen bonded turn, and S = bend. Unstructured regions are marked as U. In our study we grouped the SSE types in four categories: 1) helices (H, I); 2) 3/10 helices (G); 3) strand (E); 4) loops (B, T, S, U). Pi helices are usually attached to larger alpha helices; therefore we grouped them with helices. 3/10 helices are often poorly conserved as part of a larger loop but sometimes they are continuously linked to a larger helix and so we decided to treat them separately. B, T, S and U are grouped together because they usually belong to 3D flexible loop-like regions.

The non-redundant instance dataset is reported in Table S1 (additional file 6).

### Random structural matches

Since the aim of our study was to set up a scoring scheme and to establish accessibility and secondary structure score thresholds for discriminating true motifs among random (mostly FP) matches, we performed a pattern search using all the LM regular expressions available in the ELM database (112) in the 1037 sequences known for having at least one true annotated instance. True motif instances were filtered out from the resulting list of matches. Applying the same sequence-to-structure mapping procedure used for true motif instances, the sequences of random matches were modeled onto SCOP domains, resulting in 22,058 3D non-identical matches from 105 motifs.

### LM-specific background score distributions

Background score distributions have been obtained by scanning the 13,582 sequences of a non redundant PDB dataset (<50% sequence identity, downloaded from PDB clusters [55]) with the regular expressions of the 112 ELM motifs (total number of available motifs), mapping the 323,5412 matches onto SCOP domains and assigning them an accessibility score and a secondary structure score. Score distributions (for each feature) were then plotted for 103 LMs: score distributions for the 9 LMs with less than 10 matches are not reported. LM-specific score distributions are shown in additional file 4 (Figure S3).

### The ELM structure filter pipeline

**Figure 6:**
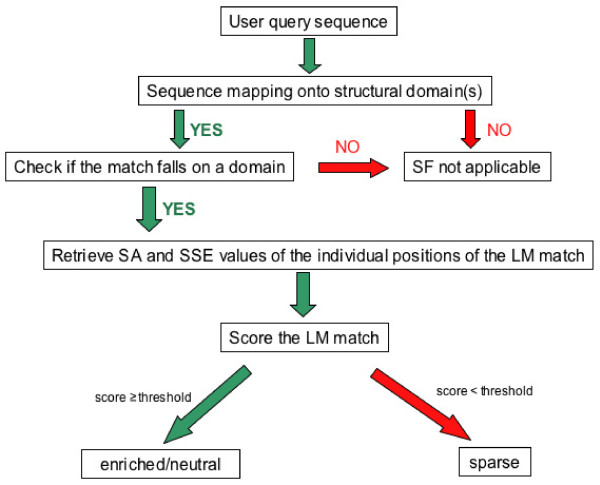
**The Structure Filter (SF) pipeline**. SA: Solvent Accessibility; SSE: Secondary Structure Element.

**Figure 7:**
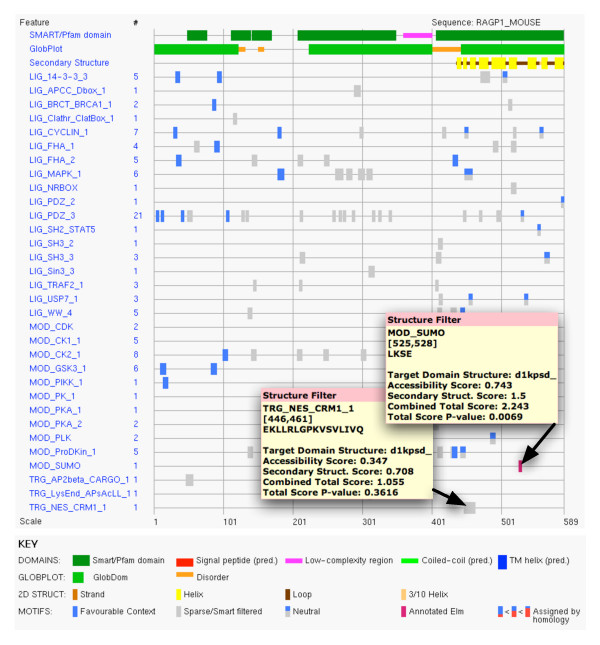
**The ELM server output page**. An example of the graphical output of the ELM structure filter for the RAGP1_MOUSE Swissprot [63] entry. The key shows the elements of the graphic. Secondary structure elements (in this case helical) are shown as yellow boxes connected by black lines (unstructured loops that tend to be surface accessible). Mouseover of the site rectangles turns on a window reporting structural information; further details of the structure filter results are available by clicking on the site rectangle.

The user query sequence submitted through either the Web Interface or the Web Service is first scanned for LM matches and then aligned to the database of ASTRAL sequences [47] derived from SCOP domains [46]. The hit with the highest sequence identity and coverage to the query sequence is selected as a reference structure. If more than one hit has the same sequence identity and coverage to the query sequence, the structure with the best experimental resolution is taken as reference and, for the same resolution, one hit is chosen randomly. This approach may result, for example, in the organism of the reference structure being different from the source organism of the user query sequence. However, since proteins with identical sequences fold into identical structures, the procedure for the selection of the reference structure does not introduce any bias in the calculation of solvent accessibility and secondary structure values. For the structure filter to be applicable, two conditions must hold: 1) the query sequence or a region of it can be aligned to one or more (non-overlapping) structural domains; 2) at least one LM match falls in an aligned region, i.e. can be mapped onto a 3D domain. The structural positions of the match are then analysed one by one and solvent accessibility and secondary structure values are collected and scored as described in Methods. The structure filter pipeline for a LM match is schematised in Figure 6 and a snapshot of the ELM server output page, displaying results of the structural filtering procedure, is reported and described in Figure 7. This latter, shows that a solved structure of the C-terminal domain of the RanGAP1 protein is available in SCOP entry d1kpsd_ that is used for structure filtering. The remainder of the sequence is filtered by the cruder domain filter. It can be observed that mouseover of the known sumoylation site reveals that it scores in the *enriched *bin and receives a significant p-value: if we did not already know it was a true motif, it would be an attractive candidate for experimental testing. Moreover, mouseover of a match to the NES motif reveals that it has poor accessibility and is assigned to the *sparse *bin. The NES motif is predominantly hydrophobic and this example, like many others falling in globular domains, is not a plausible functional site and experimental follow up would be a waste of valuable experimental effort.

### The ELM structure filter Web interface and Web service

As an initial step for feedback in the development process of the structure filter pipeline methods, the ELM structure filter functionality was implemented directly into the ELM server. This involved integration work on both the display representation in the graphical output in addition to links to the more specific details of the results.

As a second step, in order to facilitate a clean encapsulation of the structure filter pipeline code functionality and to enable future remote tool integration, a SOAP Web Service to access the functionality programmatically has been implemented and is available at .

At this link the user can find a detailed description of the web service operations and an example client implementation.

The functionality provided by the web service encompasses the current ELM server interface functionality with some additional options. For the ELM Server interface functionality, all LMs in the ELM database are matched against the query sequence and this is also the default functionality of the Web Service. The extra options implemented in the Web Service are to search the query sequence by one or more user-specified regular expressions, rather than the default ELM database regular expressions, and/or by one or more user-specified ELM identifiers from the ELM database. Where possible, to a limited extent, if the user-specified regular expression corresponds to an existing ELM this information is made known to the user.

The WSDL (Web Service Description Language) [56] file is WS-I compatible. The WS-Interoperability Basic Profile [57] proposes a set of rules to achieve interoperability of web services between different platforms. The WSDL file implements an XML document/literal style [58]. The back-end code is implemented in Java and runs on Axis2 [59] inside a Tomcat servlet container [60].

### Statistical Details

Score distributions turned out to be normal after visual inspection and quantitative Shapiro-Wilk test [61] at the 0.01 significance level. The average and standard deviation values from random match score distributions are used for the dynamical calculation of the Z-score and the corresponding one-sided p-value. The significance of the differences observed for accessibility and secondary structure frequencies in true motifs *vs *random matches was assessed through t-tests (for accessibility values) and chi-square tests (for secondary structure assignments). All the statistical calculations were performed with the R package [62].

## List of abbreviations

LM: Linear Motif; 3D: Three-dimensional; TM: True Motif; FP: False Positive; regexp: regular expression; SSE: Secondary Structure Element; S_n_: Sensitivity; S_p_: Specificity.

## Authors' contributions

AV was involved in the design and development and did most of the implementation and testing of the filter. CMG embedded the filter in the ELM resource and developed the Web Service. CG contributed to design and implementation of the pipeline. TJG was involved in the design of the filter and oversees ELM resource development. MHC oversaw the project, providing guidance through all stages of design and implementation. The manuscript was mainly authored by AV and TJG and has been read and corrected by all authors.

## Supplementary Material

Additional file 1 **Supplementary information, details and examples**. Results for true motifs and random matches taking into account all motif positions (non-wildcard + wildcard). The tables therein reported correspond to tables 1, 2, 3, 4 of the main manuscript. Moreover it describes how neglecting motif wildcard positions improved the initial benchmark set and reports the results of the manual analysis of ten nearly buried instances. Click here for file

Additional file 2 **Cumulative distributions in the case all motif position (non-wildcard + wildcard) scores are considered**. Click here for file

Additional file 3 **ROC curves**. The file contains the ROC curves for every type of score (*Q*_*acc*_, *Q*_*sse*_, *Q*_*and *_= *Q*_*acc *_+ *Q*_*sse*_) and scheme (i.e. considering both non-wildcard motif position and all motif position scores). The AUC values corresponding to ROC curves of *Q*_*acc*_(all positions), *Q*_*sse *_(all positions) and *Q*_*and *_(all positions) are 0.71, 0.67 and 0.71, respectively. Click here for file

Additional file 4 **LM-specific background score distribution plots**. Boxplots representing accessibility, secondary structure, and combined background score distributions for each ELM motif. Click here for file

Additional file 5 **Correspondences between the x-asis labels of Figure S3 (additional file**4**) and ELM names**. Click here for file

Additional file 6 **The ELM dataset of structural instances**. The complete set of 158 3D non-redundant instances from 36 different LM entries from the ELM resource release June 2007. The table reports sequence and structure information for each instance. Click here for file
