# Supplementary material for: A structure filter for the Eukaryotic Linear Motif Resource
Source: BMC Bioinformatics. 2009 Oct 24;10:351. doi: 10.1186/1471-2105-10-351 (PMC2774702; doi:10.1186/1471-2105-10-351)
Supplement: Additional file 1 — Supplementary information, details and examples. Results for true motifs and random matches taking into account all motif positions (non-wildcard + wildcard). The tables therein reported correspond to tables 1, 2, 3, 4 of the main manuscript. Moreover it describes how neglecting motif wildcard positions improved the initial benchmark set and reports the results of the manual analysis of ten nearly buried instances. [file 1471-2105-10-351-S1.DOC]

# Results based on all LM positions

The final score of a 3D prediction is the normalized sum of the single-position scores and it is calculated separately for a) all the motif positions and b) only the non-wildcard positions in the motif’s regular expression. The reason for this stems from the observation that the accessibility frequency distribution significantly differs (t-test’s confidence level = 0.99, p-value = 3.058e-05) for non-wildcard positions as opposed to wildcard positions (see Figure 3) and that, in the case of secondary structure, the frequency distribution shows a similar tendency even if the differences are not statistically significant (p-value = 0.11 for loops and helices, 0.15 for strands, 0.08 for 3/10-helices). Thus we considered the possibility that focusing on non-wildcard positions only could make the procedure more successful in discriminating true from false motif occurrences. And this was actually the case: the scores based on LM non-wildcard positions were found to be marginally more discriminating than those based on all LM positions. In the following we are presenting results for the scoring method based on all LM positions.

The score on all the positions of a motif match is calculated as:

where $N$ is the total number of the amino acid positions of a match, i.e. the number of residues in a LM occurrence, *i* is the *i*^th^ position along the match, and $q(i)$ is the positional score of position *i*.

|  |  | **loop** | **3/10 helix** | **helix** | **strand** |
| --- | --- | --- | --- | --- | --- |
| **νTM** |  | 0.69 | 0.03 | 0.20 | 0.08 |
| **νRandom** |  | 0.50 | 0.03 | 0.30 | 0.17 |
| ***νTM/ν*Random** |  | *1.38* | *1.00* | *0.67* | *0.47* |

**Table S1-1** **– Frequency of secondary structure elements observed in motifs of the benchmark and random datasets calculated considering all the LM positions.** **νTM**: frequency in the true motif instance dataset; **νRandom**: frequency in the random match dataset; $q_{\text{sse}}(i)$ = secondary structure score of position *i*.

| **bin** |  |  |  |
| --- | --- | --- | --- |
| sparse | ≤ 0.32 | ≤ 0.887 | ≤ 1.227 |
| neutral | > 0.32 and < 0.685 | > 0.887 and < 1.4 | > 1.227 and < 2.046 |
| enriched | ≥ 0.685 | ≥ 1.4 | ≥ 2.046 |

**Table S1-2**– **Accessibility and secondary structure score thresholds defining the “sparse”, “neutral” and “enriched” bins considering all the LM positions.** $Q_{\text{acc}}$: accessibility score; $Q_{\text{sse}}$: secondary structure score; $Q_{\text{and}}$**=** $Q_{\text{acc}}$+ $Q_{\text{sse}}$.

| **score type** | **bin** | **TM** | **%TM** | **random** | **%random** | **ratio** |
| --- | --- | --- | --- | --- | --- | --- |
|  | sparse | 24 | 15.19 | 8824 | 40 | 0.3798 |
|  | neutral | 86 | 54.43 | 10957 | 49.67 | 1.0958 |
|  | enriched | 48 | 30.38 | 2279 | 10.33 | 2.9409 |
|  | sparse | 32 | 20.25 | 8825 | 40 | 0.5063 |
|  | neutral | 45 | 28.48 | 8045 | 36.47 | 0.7809 |
|  | enriched | 81 | 51.27 | 5190 | 23.53 | 2.1789 |
|  | sparse | 25 | 15.82 | 8825 | 40 | 0.3955 |
|  | neutral | 85 | 53.8 | 11067 | 50.17 | 1.0724 |
|  | enriched | 48 | 30.38 | 2168 | 9.83 | 3.0905 |

**Table S1-3** – **Number and percentage of true and random motifs assigned to each bin by the different score types.** **Score type**: can be based on accessibility ($Q_{\text{acc}}$) alone, on secondary structure alone ($Q_{\text{sse}}$) or on a combination of them ($Q_{\text{and}}$**=** $Q_{\text{acc}}$+ $Q_{\text{sse}}$); **TM** (**random**): number of sparse/neutral/enriched true motif (random) matches; **%TM** (**%random**): percentage of sparse/neutral/enriched true motif (random) matches; **ratio**: %TM/%random.

| **score type** | **<S_n_>** | **<S_p_>** | **<Accuracy>** |
| --- | --- | --- | --- |
| *enriched + neutral merged* | | | |
|  | 0.843±0.018 | 0.401±0.005 | 0.404±0.005 |
|  | 0.780±0.028 | 0.403±0.006 | 0.406±0.006 |
|  | 0.836±0.012 | 0.399±0.003 | 0.402±0.003 |
| *sparse + neutral merged* | | | |
|  | 0.288±0.054 | 0.897±0.004 | 0.893±0.003 |
|  | 0.51±0.054 | 0.765±0.003 | 0.763±0.002 |
|  | 0.282±0.053 | 0.905±0.004 | 0.901±0.003 |

**Table S1-4** – **Sensitivity, specificity and accuracy averaged over the five datasets defined in the 5-fold cross validation experiment when: a) the neutral interval is incorporated into the enriched one and b) the neutral interval is incorporated into the sparse one.** <S_n_>: average sensitivity; <S_p_>: average specificity; <Accuracy>: average accuracy.

# Neglecting motif wildcard positions improved the initial benchmark set

The structure filter was initially trained and tested on the set of 173 annotated instances obtained from the ELM resource release June 2007. As explained in Methods, the procedure assigns an accessibility score and a secondary structure score to each instance. In this earlier dataset, we found 44 instances displaying a very low accessibility score. The accurate refined revision of the 173 annotated instances showed that 15 out of the 44 buried instances have questionable annotations: 11 of them (73%) had a higher accessibility value in wildcard positions than in non-wildcard positions, leading us to wonder if they might be potential false positives (FPs). A review of the literature revealed that published experimental support for these potential FPs was generally poor or inconclusive or more conclusive, unambiguous data had since become available in the literature. The instance of LIG_APCC_Dbox_1 (regular expression: .R..L..[LIVM]) matching the Aurora kinase sequence O14965 (range 370-375) was a typical example of a motif incorrectly annotated as true: the non-wildcard positions have an average relative accessibility of 0.06 as opposed to 0.4 at the wildcard positions. Furthermore, the literature is conflicting as it has also been reported that this motif is not functional in Aurora kinases but that instead they possess a functional APCC-binding motif in the intrinsically unstructured N-terminus [45]. Similar results were obtained for the instance of the LIG_Clathr_ClatBox_1 LM matching the sequence Q05140 (range 178-182); likewise, it is reported in the literature that this putative clathrin-box motif is actually not involved in the binding of the NAP domain to clathrin [46]. As a result of the structure filter prototype trials with the June 2007 ELM instances, badly annotated true motifs have been either flagged as false positives or removed in the later ELM production server releases (December 2008). Subsequently the updated dataset of 158 reliable true motif instances gave rise to better results, in terms of sensitivity and specificity, than those derived from the former dataset (data not shown).

## **Manual analysis of the ten nearly buried instances**

As explained in the main manuscript, in the benchmark dataset there are 10/158 almost completely buried true motif instances, i.e. displaying an average relative accessibility < 0.2 on the non-wildcard positions. Their manual inspection showed that, in six cases, the functional residue(s), or at least their side chains, are favourably oriented outwards from the domain surface (see additional file 1: Table_S1 and Figure 2g and 2h in the main manuscript). Three cases correspond to instances of Phospho-site (P-site) motifs (MOD_CDK, MOD_PKA_2, MOD_PKB_1), i.e. LMs encoding a phosphorylatable serine or threonine, and one is an instance of LIG_EH_1, an Asn-Pro-Phe (NPF) motif responsible for the interaction with the EH signalling domains. In the case of the buried P-sites, an allosteric effect is either known (e.g. [47] [48]) or reasonable to hypothesize, whereas the mentioned NPF motif has been classified as ambiguous in the latest ELM resource. In fact, it is almost buried in the domain and experiments supporting its function as a true motif are limited. On the other hand, several factors do not allow us to exclude that LIG_EH_1 is a true motif: a) the NF-KB protein (Q04206) matching the motif LIG_EH_1 (range 138-142) is transiently associated to the membrane, a good context to encounter an EH domain; b) as the motif is in a highly irregular 2D topology, it might be part of an allosterically regulated segment and, c) if the NPF was released, there would still be a core beta barrel that might provide a stable core fold for this domain. For these reasons, and until more data accrues, we cannot exclude that it is a true motif. True motif instances buried in a domain structural unit and becoming exposed after an allosteric conformational change, are the typical cases eluding our procedure and for which the structure filter is not in principle applicable.
