# Supplementary figures and images for: A structure filter for the Eukaryotic Linear Motif Resource
Source: BMC Bioinformatics. 2009 Oct 24;10:351. doi: 10.1186/1471-2105-10-351 (PMC2774702; doi:10.1186/1471-2105-10-351)

Cumulative distributions for all position matches

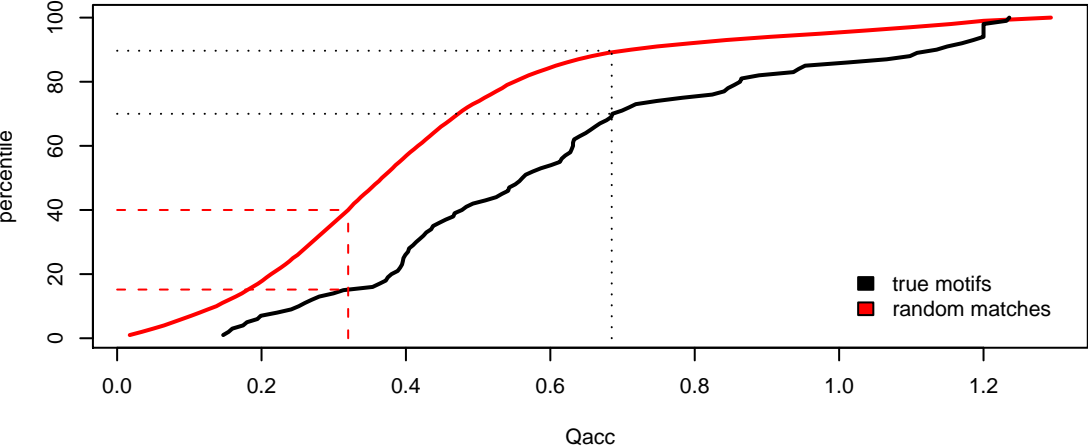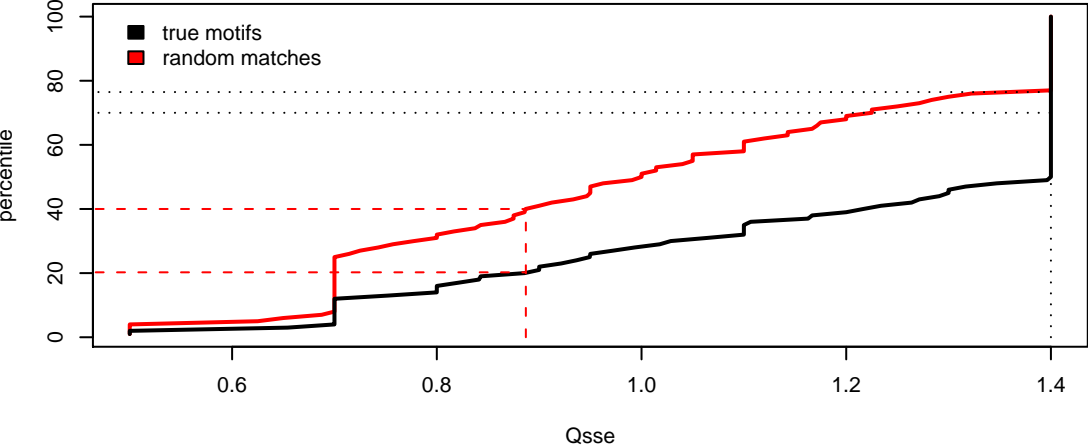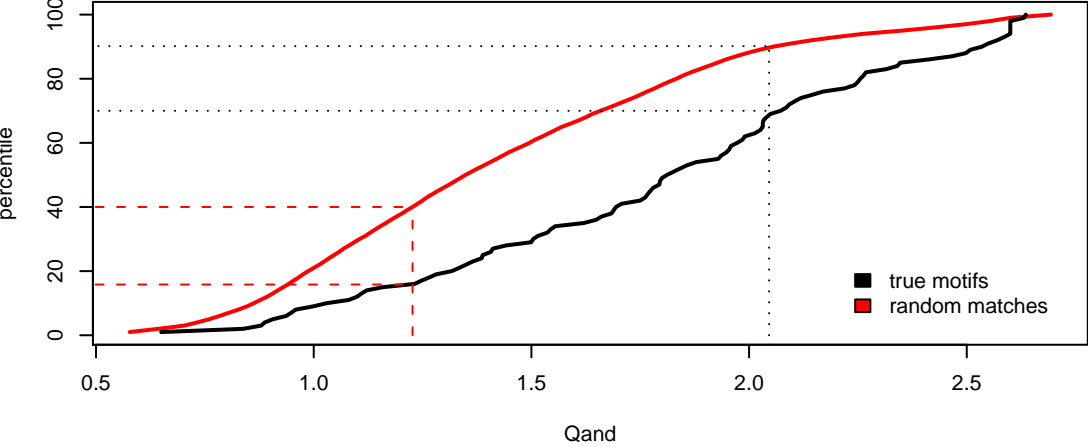

Supplement: Additional file 2 — Cumulative distributions in the case all motif position (non-wildcard + wildcard) scores are considered. [file 1471-2105-10-351-S2.PDF]

**ROC curves**

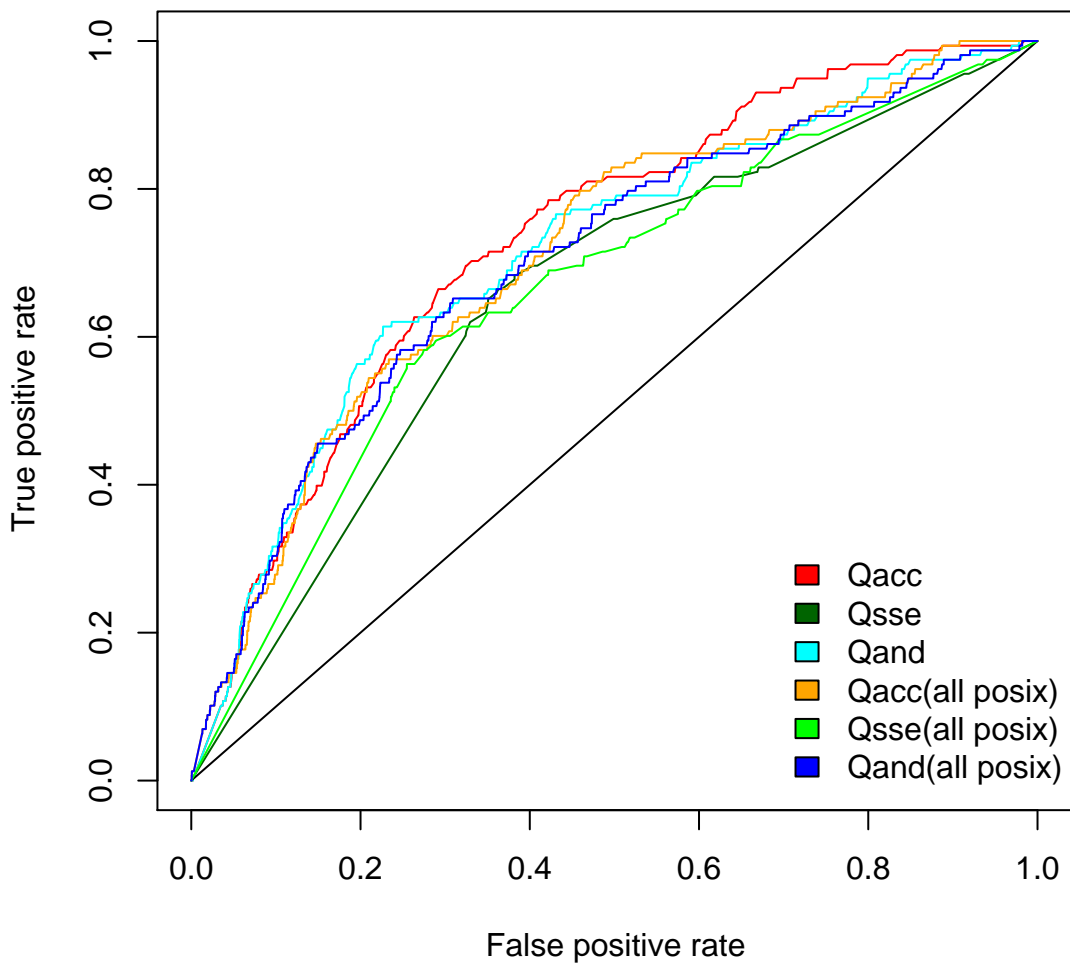

Supplement: Additional file 3 — ROC curves. The file contains the ROC curves for every type of score (Qacc, Qsse, Qand = Qacc + Qsse) and scheme (i.e. considering both non-wildcard motif position and all motif position scores). The AUC values corresponding to ROC curves of Qacc(all positions), Qsse (all positions) and Qand (all positions) are 0.71, 0.67 and 0.71, respectively. [file 1471-2105-10-351-S3.PDF]
