# Supplementary material for: A structure filter for the Eukaryotic Linear Motif Resource
Source: BMC Bioinformatics. 2009 Oct 24;10:351. doi: 10.1186/1471-2105-10-351 (PMC2774702; doi:10.1186/1471-2105-10-351)
Supplement: Additional file 4 — LM-specific background score distribution plots. Boxplots representing accessibility, secondary structure, and combined background score distributions for each ELM motif. [file 1471-2105-10-351-S4.PDF]

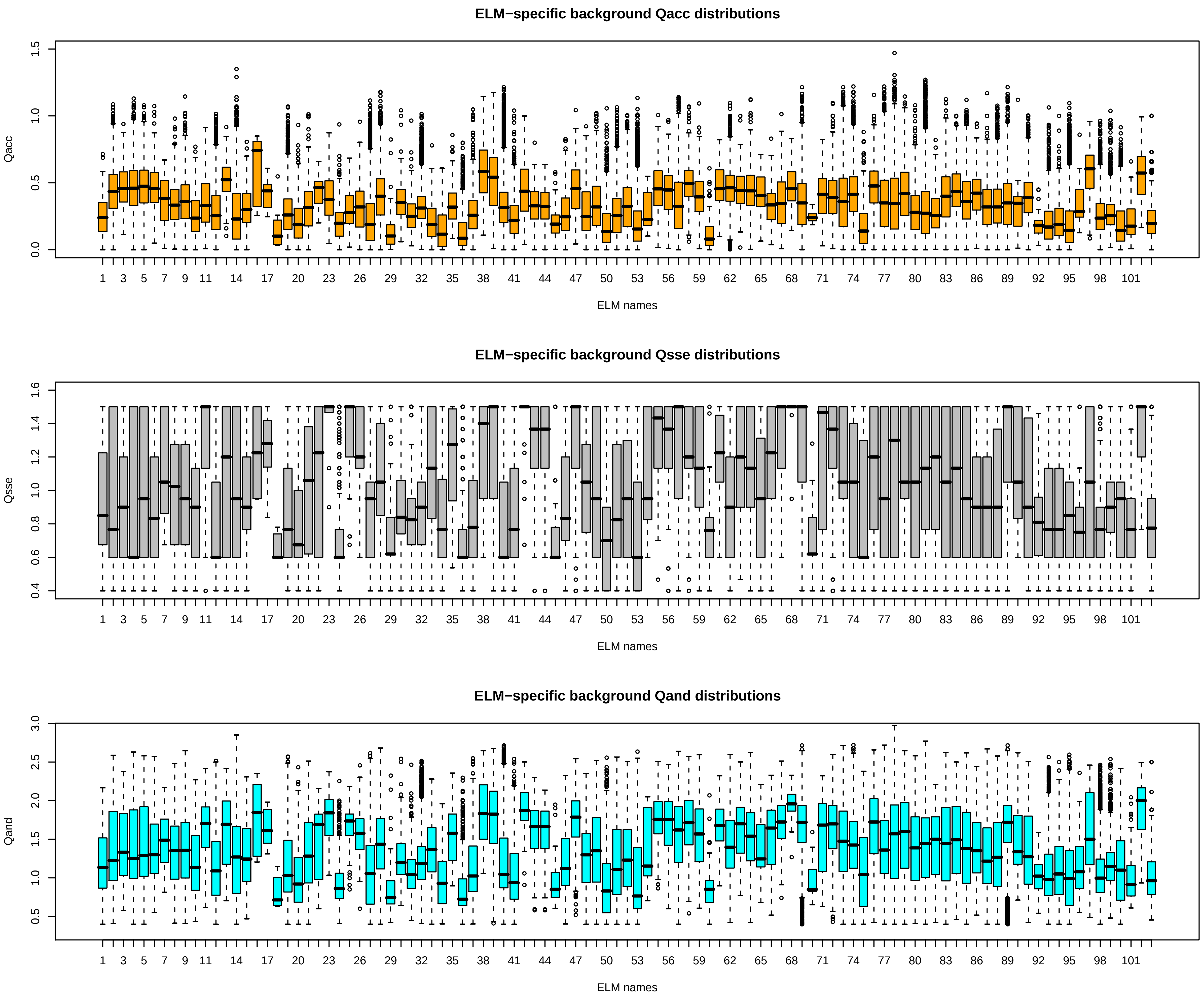

ELM−specific background Qacc distributions
Qacc
0.0
0.5
1.0
1.5
1 3 5 7 9 11 14 17 20 23 26 29 32 35 38 41 44 47 50 53 56 59 62 65 68 71 74 77 80 83 86 89 92 95 98 101
ELM names
ELM−specific background Qsse distributions
Qsse
0.4
0.6
0.8
1.0
1.2
1.4
1.6
1 3 5 7 9 11 14 17 20 23 26 29 32 35 38 41 44 47 50 53 56 59 62 65 68 71 74 77 80 83 86 89 92 95 98 101
ELM names
ELM−specific background Qand distributions
Qand
0.5
1.0
1.5
2.0
2.5
3.0
1 3 5 7 9 11 14 17 20 23 26 29 32 35 38 41 44 47 50 53 56 59 62 65 68 71 74 77 80 83 86 89 92 95 98 101
ELM names
