# Supplementary material for: A structure filter for the Eukaryotic Linear Motif Resource
Source: BMC Bioinformatics. 2009 Oct 24;10:351. doi: 10.1186/1471-2105-10-351 (PMC2774702; doi:10.1186/1471-2105-10-351)
Supplement: Additional file 5 — Correspondences between the x-asis labels of Figure S3 (additional file4) and ELM names. [file 1471-2105-10-351-S5.PDF]

| N | ELM name | N | ELM name | N | ELM name |
|---|---|---|---|---|---|
| 1 | CLV_MEL_PAP_1 | 36 | LIG_NRBOX | 71 | MOD_CAAXbox |
| 2 | CLV_NDR_NDR_1 | 37 | LIG_PCNA | 72 | MOD_CDK |
| 3 | CLV_PCSK_FUR_1 | 38 | LIG_PDZ_1 | 73 | MOD_CK1_1 |
| 4 | CLV_PCSK_KEX2_1 | 39 | LIG_PDZ_2 | 74 | MOD_CK2_1 |
| 5 | CLV_PCSK_PC1ET2_1 | 40 | LIG_PDZ_3 | 75 | MOD_CMANNOS |
| 6 | CLV_PCSK_PC7_1 | 41 | LIG_PP1 | 76 | MOD_Cter_Amidation |
| 7 | LIG_14-3-3_1 | 42 | LIG_PTAP | 77 | MOD_GSK3_1 |
| 8 | LIG_14-3-3_2 | 43 | LIG_PTB_1 | 78 | MOD_GlcNHglycan |
| 9 | LIG_14-3-3_3 | 44 | LIG_PTB_2 | 79 | MOD_N-GLC_1 |
| 10 | LIG_AP2alpha_1 | 45 | LIG_PXL | 80 | MOD_N-GLC_2 |
| 11 | LIG_AP2alpha_2 | 46 | LIG_RB | 81 | MOD_NMyristoyl |
| 12 | LIG_APCC_Dbox_1 | 47 | LIG_RGD | 82 | MOD_OFUCOSY |
| 13 | LIG_APCC_KENbox_2 | 48 | LIG_RRM_PRI_2 | 83 | MOD_PIKK_1 |
| 14 | LIG_BRCT_BRCA1_1 | 49 | LIG_SH2_GRB2 | 84 | MOD_PKA_1 |
| 15 | LIG_BRCT_BRCA1_2 | 50 | LIG_SH2_PTP2 | 85 | MOD_PKA_2 |
| 16 | LIG_BRCT_MDC1_1 | 51 | LIG_SH2_SRC | 86 | MOD_PKB_1 |
| 17 | LIG_COP1 | 52 | LIG_SH2_STAT3 | 87 | MOD_PK_1 |
| 18 | LIG_CORNRBOX | 53 | LIG_SH2_STAT5 | 88 | MOD_PLK |
| 19 | LIG_CYCLIN_1 | 54 | LIG_SH2_STAT6 | 89 | MOD_ProDKin_1 |
| 20 | LIG_Clathr_ClatBox_1 | 55 | LIG_SH3_1 | 90 | MOD_SPalmitoyl_4 |
| 21 | LIG_CtBP | 56 | LIG_SH3_2 | 91 | MOD_SUMO |
| 22 | LIG_Dynein_DLC8_1 | 57 | LIG_SH3_3 | 92 | MOD_TYR_ITAM |
| 23 | LIG_EH | 58 | LIG_SH3_4 | 93 | MOD_TYR_ITIM |
| 24 | LIG_EH1 | 59 | LIG_SH3_5 | 94 | MOD_TYR_ITSM |
| 25 | LIG_EVH1_I | 60 | LIG_Sin3_3 | 95 | TRG_ENDOCYTIC_2 |
| 26 | LIG_EVH1_II | 61 | LIG_TNKBM | 96 | TRG_ER_diArg_1 |
| 27 | LIG_FHA_1 | 62 | LIG_TRAF2_1 | 97 | TRG_ER_diLys_1 |
| 28 | LIG_FHA_2 | 63 | LIG_TRAF2_2 | 98 | TRG_LysEnd_APsAcLL_1 |
| 29 | LIG_HP1_1 | 64 | LIG_TRAF6 | 99 | TRG_NES_CRM1_1 |
| 30 | LIG_IQ | 65 | LIG_ULM_U2AF65_1 | 100 | TRG_PEX |
| 31 | LIG_MAD2 | 66 | LIG_WRPW_2 | 101 | TRG_PML_SV |
| 32 | LIG_MAPK_1 | 67 | LIG_WW_1 | 102 | TRG_PTS1 |
| 33 | LIG_MAPK_2 | 68 | LIG_WW_2 | 103 | TRG_PTS2 |
| 34 | LIG_MDM2 | 69 | LIG_WW_4 | | |
| 35 | LIG_MYND | 70 | MOD_ASX_betaOH_EGF | | |

**Table S2** – Correspondences between the x-asis labels of Figure S3 and ELM names. N is the number reported on the Figure S3 x-axis.
